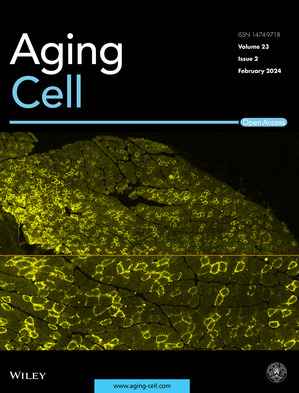# Additional Cover

**DOI:** 10.1111/acel.14117

**Published:** 2024-02-12

**Authors:** Alaa Elgaabari, Nana Imatomi, Hirochika Kido, Takashi Nakashima, Shoko Okuda, Yoshitaka Manabe, Shoko Sawano, Wataru Mizunoya, Ryuki Kaneko, Sakiho Tanaka, Takahiro Maeno, Yuji Matsuyoshi, Miyumi Seki, So Kuwakado, Kahona Zushi, Nasibeh Daneshvar, Mako Nakamura, Takahiro Suzuki, Kenji Sunagawa, Judy E. Anderson, Ronald E. Allen, Ryuichi Tatsumi

## Abstract

Cover legend: The cover image is based on the Research Article *Age‐related nitration/dysfunction of myogenic stem cell activator HGF* by Alaa Elgaabari et al., https://doi.org/10.1111/acel.14041